# Emergency Surgery

**Published:** 1910-07

**Authors:** 


**Emergency Surgery.** By **John W. Sluss,** M.D., Professor of Anatomy, Indiana University School of Medicine, Indianapolis. Second edition. 12mo., pp. 762. Illustrated. Philadelphia: P. Blakiston's Son & Co. (Cloth, $3.50.)

For a medical man not skilled in surgery, this book is both convenient and valuable. A study of it will prepare him to meet emergencies as they arise. The first edition was exhausted within a year which shows the demand for a work of this kind. The author has added a chapter on the general technic of laparatomy; also some new matter on the subjects of pericardiotomy and subsplenic abscess. Additions have also been made to the technic of spinal anesthesia. The arrangement of the book is such that the reader can investigate any subject he desires without examining previous chapters. J. A. R.

